# Phenotype, Polyfunctionality, and Antiviral Activity of *in vitro* Stimulated CD8^+^ T-Cells From HIV^+^ Subjects Who Initiated cART at Different Time-Points After Acute Infection

**DOI:** 10.3389/fimmu.2018.02443

**Published:** 2018-10-23

**Authors:** Jimena Salido, María Julia Ruiz, César Trifone, María Inés Figueroa, María Paula Caruso, María Magdalena Gherardi, Omar Sued, Horacio Salomón, Natalia Laufer, Yanina Ghiglione, Gabriela Turk

**Affiliations:** ^1^Consejo Nacional de Investigaciones Científicas y Técnicas (CONICET)-Universidad de Buenos Aires, Instituto de Investigaciones Biomédicas en Retrovirus y Sida (INBIRS), Buenos Aires, Argentina; ^2^Fundación Huésped, Buenos Aires, Argentina; ^3^Hospital General de Agudos “Dr. JA Fernández”, Buenos Aires, Argentina

**Keywords:** HIV functional cure, expanded CD8^+^ T-cell response, polyfunctionality and phenotype, antiviral activity, time of cART initiation

## Abstract

Since anti-HIV treatment cannot cure the infection, many strategies have been proposed to eradicate the viral reservoir, which still remains as a major challenge. The success of some of these strategies will rely on the ability of HIV-specific CD8^+^ T-cells (CD8TC) to clear reactivated infected cells. Here, we aimed to investigate the phenotype and function of *in vitro* expanded CD8TC obtained from HIV^+^ subjects on combination antiretroviral therapy (cART), either initiated earlier (median = 3 months postinfection, ET: Early treatment) or later (median = 20 months postinfection, DT: Delayed treatment) after infection. Peripheral blood mononuclear cells from 12 DT and 13 ET subjects were obtained and stimulated with Nef and Gag peptide pools plus IL-2 for 14 days. ELISPOT was performed pre- and post-expansion. CD8TC memory/effector phenotype, PD-1 expression, polyfunctionality (CD107a/b, IFN-γ, IL-2, CCL4 (MIP-1β), and/or TNF-α production) and antiviral activity were evaluated post-expansion. Magnitude of ELISPOT responses increased after expansion by 10^3^ times, in both groups. Expanded cells were highly polyfunctional, regardless of time of cART initiation. The memory/effector phenotype distribution was sharply skewed toward an effector phenotype after expansion in both groups although ET subjects showed significantly higher proportions of stem-cell and central memory CD8TCs. PD-1 expression was clustered in HIV-specific effector memory CD8TCs, subset that also showed the highest proportion of cytokine–producing cells. Moreover, PD-1 expression directly correlated with CD8TC functionality. Expanded CD8TCs from DT and ET subjects were highly capable of mediating antiviral activity, measured by two different assays. Antiviral function directly correlated with the proportion of fully differentiated effector cells (viral inhibition assay) as well as with CD8TC polyfunctionality and PD-1 expression (VITAL assay). In sum, we show that, despite being dampened in subjects on cART, the HIV-specific CD8TC response could be selectively stimulated and expanded *in vitro*, presenting a high proportion of cells able to carry-out multiple effector functions. Timing of cART initiation had an impact on the memory/effector differentiation phenotype, most likely reflecting how different periods of antigen persistence affected immune function. Overall, these results have important implications for the design and evaluation of strategies aimed at modulating CD8TCs to achieve the HIV functional cure.

## Introduction

Infection with Human Immunodeficiency Virus (HIV) causes an irreversible and profound deterioration of the immune system as well as abrogated T cell homeostasis, ultimately leading to the development of acquired immunodeficiency syndrome (AIDS) in the vast majority of infected persons. Despite dramatic advances made over the past three decades, it still constitutes a major public health concern worldwide. Following virus transmission, acute/early phase of infection is characterized by a high-level peak of viremia, rapid loss of CD4^+^ T-cells in both peripheral blood and mucosal lymphoid tissues, and clinical symptoms (1–3). Emergence of HIV-specific CD8^+^ T-cell response is associated with the drop of plasma viremia to a stable level, known as the viral set-point (4).

Since the implementation of combination antiretroviral therapy (cART), AIDS-associated death rates have decreased drastically, and morbidity and mortality of HIV^+^ subjects have been considerably reduced (with a concomitant improvement in their quality of life). Besides, transmission risks have diminished (with an impact on the global epidemic dynamics) (5). cART can quickly and persistently suppress viral replication but, if treatment is interrupted, plasma viral load (VL) rapidly increases (6, 7). The failure to eradicate HIV infection is due to the intrinsic stability of the viral genome in latently infected CD4^+^ T-cells and also other long-lived cells (8). Consequently, cART is currently a lifelong treatment with some limitations: the need of daily doses, the development of viral resistance, toxicity, and the impossibility to clear the infection. Moreover, even effectively treated HIV-infected individuals have a greater risk of experiencing non-AIDS related morbidity and mortality events than age-matched HIV-uninfected adults (including accelerated immune aging, higher cardiovascular risk, coagulopathies and other metabolic disorders), indicating that even effective cART cannot fully restore health (9). In this line, the idea of developing a cure for HIV infection has gained intense interest over the last years; this is the development of a therapeutic intervention or approach that controls (functional cure or long-term remission in the absence of cART) or eliminates (sterilizing cure) HIV infection (3, 10). To pursue this objective, several strategies are being investigated as reviewed in Deeks et al. (3) and Pitman et al. (11). Many of these strategies, such as the “shock and kill” model, require the preexistence of cellular responses being able to actively clear reactivated and/or persistent virus. However, CD8^+^ T-cell responses are severely waned in subjects on cART, limiting thus these approaches (12).

During the natural course of infection, HIV-specific CD8^+^ T-cells play a central role in the control of viral replication, particularly during acute infection (2, 4, 12). In this setting, our group has provided evidence regarding different qualitative aspects of the response (specificity, functionality, and phenotype) that better associate with virus control in an acute infection cohort from Argentina (13, 14). Progressive infection is characterized by the presence of a narrow, oligofunctional and exhausted cellular response. As subjects initiate cART, the magnitude of the CD8^+^ T-cell response declines rapidly (15–20) though some publications have demonstrated that the remaining cells still possess antiviral functions (21–23). Importantly, it has been recently illustrated in a non-human primate model that CD8^+^ T-cells might have a role in controlling viral production even on cART (24). Moreover, some studies have indicated that CD8^+^ T-cells still exert selective pressure in subjects on cART, suggesting that this response might be acting on viral residual replication as reviewed in McIlroy (25). Finally, different attributes of the CD8^+^ T-cell response have been related to the reservoir size once the subjects are on treatment (26–30). However, cART interruption leads to rapid viral rebound, indicating that control mediated by CD8^+^ T-cells is temporal and inefficient (31).

Thus, the success of the strategies aimed at eliminating the viral reservoir using HIV-specific memory CD8^+^ T-cells that persist after cART initiation will require refined knowledge about the functional and phenotypic properties of these cells. Here, we aimed to investigate the phenotype and function of *in vitro* expanded CD8^+^ T-cells from HIV^+^ subjects on cART who initiated treatment either early or late after infection. Results indicated that HIV-specific cells can be selectively stimulated and expanded *in vitro*, presenting a high proportion of cells able to carry out multiple effector functions. On the contrary, cART initiation timing had an impact on the memory/effector differentiation phenotype. Despite this, expanded cells from both groups had potent antiviral activity. Thus, we propose that despite differences in the duration of antigen persistence, HIV-specific CD8^+^ T-cells remain detectable and functional but should be boosted in order to support viral clearance.

## Materials and methods

### Study subjects

Twenty-five HIV^+^ subjects were enrolled during acute/early infection and followed up longitudinally as part of the *Grupo Argentino de Seroconversión* study group. Enrollment criteria were described elsewhere (32, 33). Twelve subjects initiated cART after 4 months since the estimated date of infection (from now on Delayed Treatment (DT) group), and 13 initiated cART within 4 months post-infection (Early Treatment group, ET). For this study, samples were collected from study participants at ~12 months post-cART initiation.

This study was reviewed and approved by two institutional review boards: *Comité de Bioética Humana, Fundación Huésped*, and *Comité de Ética Humana, Facultad de Medicina, Universidad de Buenos Aires*, Buenos Aires, Argentina. All participants provided written informed consents and agreed to participate in this study in line with the Declaration of Helsinki.

### Samples

Forty ml of whole blood were collected from study participants, centrifuged to separate plasma, and stored at −80°C. Peripheral blood mononuclear cells (PBMCs) were isolated by Ficoll-Hypaque density gradient centrifugation (Amersham, Sweden) and cryopreserved for subsequent functional assays. Plasma viral load (VL) was determined by branched-DNA, Versant HIV-1 RNA 3.0 assay (Siemens Healthcare, UK). CD4^+^ and CD8^+^ T-cell counts were determined using TruCount absolute-count tubes (BD Bioscences, USA) on a BD FACSCalibur flow cytometer.

### Peptide pools

Potential T-cell epitope (PTE) peptide panels corresponding to Nef (unique pool, *n* = 127 peptides) and Gag [2 pools: p17 (*n* = 97) and p24 (*n* = 128)] HIV proteins and the cytomegalovirus (CMV), Epstein-Barr virus, and influenza virus (CEF) peptide pool were obtained from the NIH AIDS Reagent Program (34, 35). Lyophilized peptides were dissolved in dimethyl sulfoxide (DMSO) at 40 μg/μl and stored at −20°C.

### HIV-specific CD8^+^ T-cell expansion

**Figure 1:**
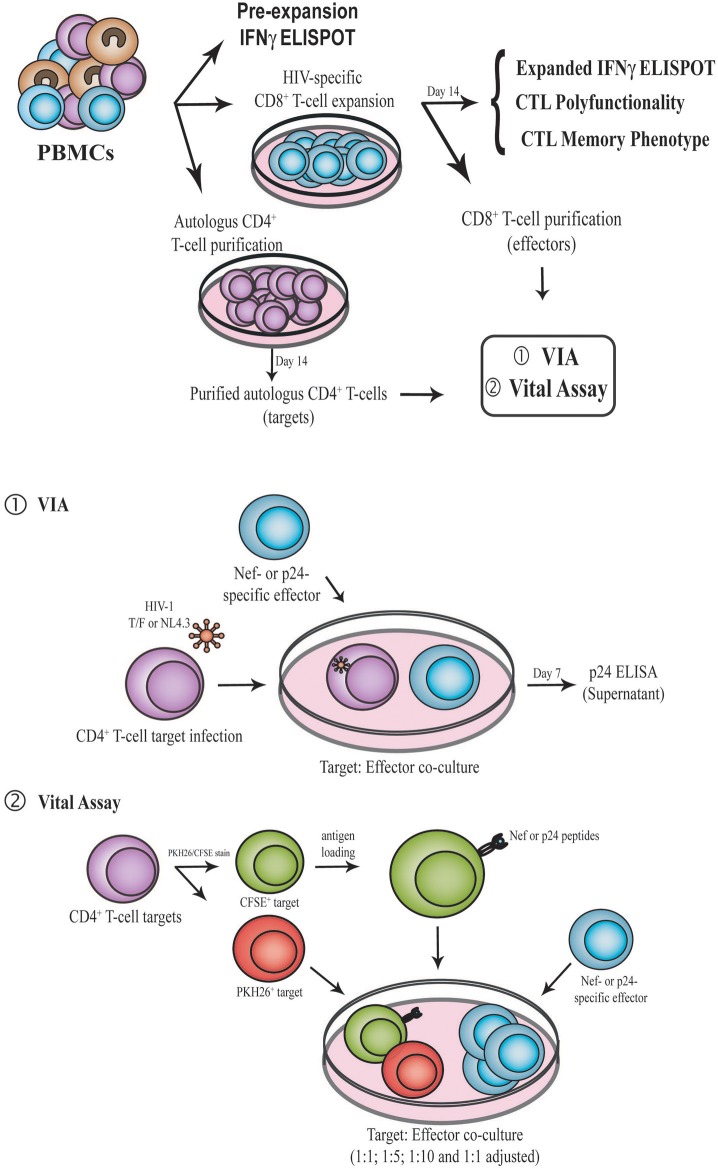
Schematic representation of the experimental design. PBMC samples were obtained from enrolled subjects. First, IFN-γ ELISPOT was performed directly *ex vivo* (pre-expansion). PBMCs were then expanded for 14 days in the presence of Nef or p24 peptide pool, generating Nef-specific and p24-specific effector. At day 14, IFN-γ ELISPOT, CD8^+^ T-cell polyfunctionality and CD8^+^ T-cell phenotype (CCR7, CD45RO, CD95, and PD-1) were evaluated by flow cytometry. Expanded CD8^+^ T-cells were also purified and used as effectors in the VIA (1) and VITAL (2) assays. In parallel, autologous CD4^+^ T-cells were purified with CD3.8 bispecific antibodies to generate CD4^+^ target cells. See M&M for further details. PBMC, Peripheral blood mononuclear cells; VIA, Viral inhibition assay; CTL, CD8^+^ T-lymphocyte.

Cryopreserved PBMCs were thawed in PBS (Sigma-Aldrich), 2% fetal bovine serum [FBS; Gibco BRL], and 1 mM EDTA supplemented with 25 U/ml DNase I (Benzonase nuclease; Sigma-Aldrich) and then rested overnight (ON) in DNase-free complete RPMI medium (RPMIc; RPMI 1640 [Gibco BRL], 10% fetal bovine serum [FBS; Gibco BRL], 2 mM L-glutamine [Gibco BRL], 100 U/ml penicillin [Gibco BRL], 100 μg/ml streptomycin [Gibco BRL], 10mM HEPES [Gibco BRL]). Rested PBMCs were cultured in 12-well plates at a density of 2–3 × 10^6^ cells/ml in cRPMI medium supplemented with 100 U/ml IL-2 (Biolegend Inc, USA) and in the presence of 1 μg/ml of the corresponding HIV peptide pool or CEF peptide pools, for 14 days. Medium was replaced every 72 h with freshly-prepared cRPMI supplemented with IL-2. Expanded cells were subsequently studied by ELISPOT, flow cytometry, and antiviral activity assays (Figure 1).

### ELISPOT assay

Interferon gamma (IFN-γ)-secreting cells were evaluated by ELISPOT before and after HIV-specific CD8^+^ T-cell expansion (pre-expansion, i.e., immediately after PBMC ON rest, and post-expansion) following the protocols published previously (13, 36, 37).

### Flow cytometry

CD8^+^ T-cell phenotype and functionality were evaluated in 14-day expanded cells by flow cytometry as reported by our group, with modifications (13, 14, 38). Briefly, cells were re-stimulated with the designated peptide pool (same used for expansion, at 2 μg/ml) or DMSO (to account for background) plus costimulatory antibodies (anti-CD28 and anti-CD49d; 1 μg/ml; BD Biosciences), monensin (Golgistop, 0.7 μl/ml; BD Biosciences) and brefeldin A (10 μg/ml; BD Biosciences) for 5 h at 37°C. Anti-CD107a/b-FITC antibodies (BD Biosciences) were also added to identify degranulating cells. For the functionality panel, cells were stained upon stimulation with Zombie NIR™ Fixable Viability Kit (Biolegend, USA), and the following conjugated antibodies: anti-CD14-V450, anti-CD19-V450, anti-CD3-BV786, anti-CD8-APC, and anti-CD4-BV650 (BD Biosciences). Then, cells were permeabilized (Permeabilization Wash Buffer, Biolegend), fixed (Fixation Buffer, Biolegend), and subsequently stained using anti-IL-2–PerCP-Cy5.5, anti-TNF-α-PECy7, anti-IFN-γ-BV711, and anti-CCL4-PE conjugated antibodies (BD Biosciences).

In parallel, CD8^+^ T-cell memory phenotype was studied. Cells were shortly stimulated as described above and afterwards stained with Zombie NIR™ Fixable Viability Kit plus the following conjugated antibodies: anti-CCR7-Alexa700, anti-PD-1-PE, anti-CD3-BV786, anti-CD8-APC, anti-CD4-BV650, anti-CD14-V450, anti-CD19-V450, anti-CD45RO-PerCPCy5.5, and anti-CD95-PE-CF594 (BD Biosciences, USA). Then, cells were permeabilized, fixed and stained with anti-IL-2, anti-TNF-α, and anti-IFN-γ antibodies, all of them conjugated to FITC (BD Biosciences) to identify specific cells regardless of its function.

Flow cytometry data acquisition was performed on a 3-laser 14-color BD FACSAria FUSION flow cytometer using the BD FACSDiva v 8.0.1 software (BD Biosciences). Instrument settings and fluorescence compensation were performed using unstained samples and single stained BD CompBeads (BD Bioscience). Isotype controls, consisting of expanded, stimulated cells stained with conjugated antibodies to CD14, CD19, CD3, CD4, and CD8 surface molecules plus the isotype controls corresponding to the CCR7, CD45RO, CD95, PD-1, and/or the corresponding intracellular marker staining, were performed for each individual in order to set negative populations accurately.

Acquired data was analyzed using FlowJo v10 (Data Analysis Software, LLC). Gating strategy was performed as shown in Figure S1. First, single cells were selected in a forward scatter area (FSC-A) vs. FSC-Height plot. Then, dead cells were excluded on the bases of Zombie NIR™ fluorescence and monocytes as well as B lymphocytes were also excluded on the bases of V450 fluorescence (CD14 and CD19 staining). Subsequently, the lymphocyte population was selected in a FSC-A vs. side scatter (SSC) plot. Samples with at least 100,000 events in the lymphocyte gate were included in subsequent analyses. Finally, CD3^+^ CD8^+^ (or CD4^+^) cells were gated in CD3-vs.-CD8 (or CD4) dot plots.

To study CD8^+^ T-cell polyfunctionality, CD8 vs. CD107a/b, IFN-γ, IL-2, CCL4, or TNF-α plots were constructed. After the gates for each function were created, the Boolean gate platform was used to create the full array of obtainable combinations, equating to 32 possible combinations. Data presented correspond to background-subtracted results using the DMSO plus CD28/CD49d stimulation. This was performed on a cytokine-subset-by-cytokine-subset basis, i.e., subtracting the result from this condition for a given cytokine subset to the same subset of a peptide-stimulated condition. One standard deviation (SDs) above background was set as the threshold for determining positive responses. Values below this threshold were set at 0.

For phenotype analysis, HIV-specific CD8^+^ T-cells were identified in a CD8 vs. FITC plot (CD107a/b, IFN-γ, IL-2, CCL4, and TNF-α). A positive cytokine response was defined as at least twice the background value, >0.05% after subtraction of background and at least 1,000 events. This criterion was established to minimize the possibility of error due to a low number of events when further subdividing these cells into the different memory subsets. To analyze the distribution of the different phenotype subsets, CD45RO vs. CCR7 density plots were constructed on both bulk and HIV-specific CD8^+^ T-cells to identify central memory T-cells (T_CM_, CCR7^+^/CD45RO^+^), effector memory T-cells (T_EM_, CCR7^−^/CD45RO^+^), and terminal effector T-cells (T_TE_, CCR7^−^/CD45RO^−^). CD95 expression was analyzed within the CD45RO^−^CCR7^+^ cells thus defining naïve T-cells (T_N_, CCR7^+^/CD45RO^−^/CD95^−^) and stem-cell memory T-cells (T_SCM_, CCR7^+^/CD45RO^−^/CD95^+^). Additionally, PD-1 expression was studied both on bulk (total and also within each memory subpopulations) and HIV-specific CD8^+^ T-cells.

### CD8^+^ T-cell antiviral activity

Antiviral activity of expanded CD8^+^ T-cells was evaluated by two different assays (Figure 1): The Viral Inhibition Assay [VIA, (13, 39, 40)] and the VITAL assay [adapted from Hermans et al. (41)]. In the former, both cytolitic and non-cytolitic mechanisms of viral inhibition are accounted while in the latter direct cell-mediated toxicity is measured. For both assays, target (autologous CD4^+^ T-cells) and effector (expanded CD8^+^ T-cells) were prepared following the same procedure:

#### Generation and isolation of expanded Nef- and p24-specific effector CD8^+^ T-cells

PBMCs were thawed and stimulated with either peptide pools spanning Nef or p24 proteins as stated above (day 0). At day 13 post-expansion and after a 5-h short re-stimulation (as described previously), percentages of cytokine-producing cells and degranulating cells (i.e., total HIV-specific cells) were assessed by flow cytometry. At day 14, expanded CD8^+^ T-cells were isolated by positive selection using Anti-Human CD8 Magnetic Particles (BD Biosciences).

#### Generation of autologous CD4^+^ T-cell targets

PBMCs were thawed and cultured in cRPMI medium supplemented with 50 U/ml IL-2 plus 0.5 μg/ml CD3/8 bi-specific antibody (obtained through the NIH AIDS Reagent Program, Division of AIDS, NIAID, NIH: Anti-Human CD3/8 Bi-specific Monoclonal from Drs. Johnson Wong and Galit Alter). PBMC treatment with CD3/8 bi-specific antibody results in the elimination of CD8^+^ T-cells and purification of activated CD4^+^ T-cells. To determine purity, expanded CD4^+^ T-cell were stained with Zombie viability kit, anti-CD3-PECy7, anti-CD4-PerCP, and anti-CD8-BV510 at day 13. In all cases, 90% purity was achieved.

#### Viral inhibition assay (VIA) (Figure 1)

Target cells (purified and activated CD4^+^ T-cells) were infected in parallel with 4 different viral strains: an X4-tropic laboratory strain (NL4-3) and three HIV-1 clade B transmitted/founder (T/F) primary viruses selected from the full panel of T/F Infectious Molecular Clones available at the NIH AIDS Reagent program (Division of AIDS, NIAID, NIH: Cat #11742 (X4-tropic T/F virus, from now on Virus 4, V4), cat #11746 (R5-tropic T/F virus obtained after an event of heterosexual transmission, from now on Virus 8, V8), and Cat #11749 (R5-tropic T/F virus obtained after an event of male-to-male transmission, from now on Virus 9, V9) from Dr. John Kappes (42–45). Pseudotyped viral stocks were produced by co-transfecting 293T cells with the corresponding HIV plasmid together with a plasmid encoding the Vesicular Stomatitis Virus (VSV) protein G, using the X-treme GENE 9 DNA transfection reagent (Roche, Switzerland). Culture supernatants were harvested 48 h post-transfection clarified by centrifugation at 600 g for 15 min at 4°C, fractioned and stored at −80°C until use. Viral titer was estimated by p24 antigen quantitation by ELISA (Sino Biological Inc., China).

Viruses were added to target cells at 15 ng p24/100,000 CD4^+^ T-cells. In order to improve infection efficiency, plates were first centrifuged at 1,200 g for 1 h at 22°C (spinoculation), and then viral adsorption was let to proceed for an extra hour at 37°C in a humidified CO_2_ incubator. After infection, cells were washed twice and co-cultured at 1:1 ratio with purified expanded Nef-specific or p24-specific CD8^+^ T-cells (effectors) in U-bottom 96-well plates, in cRPMI medium containing 10 U/ml IL-2. Infectivity controls consisted of infected CD4^+^ T-cell targets without CD8^+^ T-cell effectors. Uninfected controls consisted of uninfected target cells without effectors. All conditions were assayed in triplicate. At day 4 post-infection, half of the culture supernatant was removed and replenished with fresh medium. At day 7, supernatants were collected and stored at −20°C until p24 antigen quantitation by ELISA. CD8^+^ T-cell anti-HIV suppressive capacity was calculated as the log_10_ of the percentage of p24 antigen loss when CD8^+^ T-cells were present in the culture compared to CD4^+^ T-cell infected controls without effectors, for each virus.

#### *In vitro* cell killing assay (VITAL assay, Figure 1)

2 × 10^6^ cells/ml target cells (purified and activated CD4^+^ T-cells) were first stained with 1 μM of CFSE (Molecular Probes, USA) at 37°C for 8 min, followed by the addition of an equal volume of FBS to quench the reaction. After CFSE staining, cells were loaded with peptide antigens by incubation for 2 h in cRPMI supplemented with 2 μg/ml of either Nef or p24 peptide pool. In parallel, untreated CD4^+^ T-cells (i.e., not loaded with peptides) were labeled with 2 μM PKH26 red fluorescent cell linker (Sigma-Aldrich, USA). Peptide-loaded CFSE^+^ and unloaded PKH26^+^ cells were extensively washed, combined and plated in U-bottomed 96-well plates at 5 × 10^4^ cells of each fluorescent population per well. Nef- or p24-specific effector cells were added to the corresponding wells at the indicated target-to-effector (T:E) ratios (1:1, 1:5, 1:10 and 1:1 adjusted [i.e., 1:1 ratio adjusted to proportion of specific CD8^+^ T-cell effectors as determined by intracellular staining and flow cytometry]), in triplicate. Following overnight incubation at 37°C, cells were stained with Zombie Viability Kit and anti-CD3-PECy7, anti-CD4-PerCP, and anti-CD8-BV510 antibodies, and analyzed in a 2-laser, 8-color BD FACSCanto flow cytometer. Data acquisition was performed using the BD FACSDiva software and analyzed subsequently with FlowJo v10 software (Data Analysis Software, LLC). Initial gating was performed in a FSC-A *vs*. FSC-H plot to exclude doublets, and then lymphocytes were selected in a FSC-H vs. SSC-A plot. Subsequently, Zombie- negative cells (i.e., living cells) were gated. Then, CD3^+^CD4^+^ cells were selected and the analysis was performed on this population. CFSE vs. PKH26 plots were constructed, and cell survival of antigen-loaded targets cells (CFSE-positive) in the presence of effectors was determined compared to conditions without effector cells. Adjusted survival was calculated as the mean percentage of CFSE^+^ events with added effector *vs*. the condition with no effectors. Finally, the percentage of specific lysis was calculated using the equation: %specific lysis: 100—%adjusted survival.

### Data analysis

The presumed date of infection was estimated as reported previously (32, 33). Date of cART initiation was informed by clinicians. Most data was expressed as median values with interquartile ranges (25 to 75%, IQ25-75) and analyzed by nonparametric methods using GraphPad Prism 7 software, unless otherwise stated. Inter- and intra-group comparisons were performed using Mann-Whitney and Wilcoxon tests, respectively. Correlation analyses were performed using the Spearman's rank test. In this case, *p*-values were adjusted for multiple comparisons using a false discovery rate (FDR) procedure, according to the Benjamini and Hochberg method, using the GraphPad Prism 7 software.

For the polyfunctionality analysis and cell phenotype data sets generated by flow cytometry, SPICE 6.0 software (https://niaid.github.io/spice/) was used following the experimental and technical considerations published by the software developers (46). In particular, Student's *t*-test and a partial permutation test were used to compare distribution profiles between groups.

All tests were considered significant when the *p*-value was <0.05. Adjusted *p*-values for correlation analyses were considered significant when <0.1.

## Results

### Study subjects

Twenty-five HIV^+^ subjects were enrolled during acute/early HIV infection (within 6 months from presumed date of infection) and followed up for over at least 1 year after cART initiation. Subjects were segregated into two subgroups according to cART initiation timing. A 4-month post-presumed date of infection cut-off was selected based on the fact that CD8^+^ T-cells are key players in viral set point establishment, which is usually reached around 4 months post-infection (2, 47). Thus, the responses evaluated would represent pre- and post-set-point scenarios. Delayed Treatment group (DT; 12 subjects) included subjects who initiated treatment after 4 months from presumed date of infection (median time to cART initiation = 20 months, [Interquartile range IQ25-75: 9.5–24.25 months]) while Early Treatment group (ET; 13 subjects) included subjects who started cART within 4 months post-presumed date of infection (median time to cART initiation = 3 months [IQ 25–75: 2–3.5 months]). All samples used for the study were obtained at ~1 year after treatment initiation (DT: median time on cART = 13 months [IQ25-75: 5.75–18 months]; ET: median = 14 months [IQ25-75: 12–16.5 months]). Detailed descriptions of participants are shown in Table 1.

**Table 1:** Clinical data corresponding to HIV^+^ subjects enrolled per study group.

				**CD4 count**		**CD4/CD8**	
**ID^a^**	**Time to cART (months)^b^**	**Time on cART (months)^c^**	**Pre-treatment Viral Load^d^^,^^e^**	**Before cART^d^^,^^f^**	**On cART^f^^,^^g^**	**Before cART^d^^,^^f^**	**On cART^f^^,^^g^**
DT1	50	22	48526	383	524	0.72	1.35
DT2	22	18	132317	324	297	0.46	0.52
DT3	8	18	125716	391	711	0.48	0.65
DT4	11	5	27244	465	535	0.45	0.74
DT5	25	5	4612	408	739	0.48	1.00
DT6	19	15	66929	246	501	0.24	0.62
DT7	50	5	15297	330	468	0.42	0.77
DT8	5	12	>500000	279	528	0.26	0.72
DT9	21	14	28288	184	579	0.30	0.88
DT10	13	20	1702	357	563	0.44	0.72
DT11	22^*^	12	10033	819	819	0.88	1.36
DT12	9	8	111893	629	880	0.85	1.53
ET1	2	14	>500000	627	594	0.66	0.89
ET2	4	18	283441	187	522	0.20	0.88
ET3	1	11	36338	768	1099	1.18	1.83
ET4	3	14	17292	612	1342	0.33	1.32
ET5	3	15	20106	787	829	1.06	1.69
ET6	2	18	>500000	421	854	0.31	1.89
ET7	1	13	>500000	435	942	0.13	1.52
ET8	3	12	172468	435	657	0.59	1.16
ET9	3	17	26547	400	728	0.70	1.79
ET10	3^*^	11	231360	330	1080	0.18	1.24
ET11	2	12	>500000	654	1026	0.42	1.47
ET12	4	16	>500000	339	691	0.08	0.66
ET13	4	12	>500000	213	386	0.19	0.81

a *DT, Delayed Treatment Group; ET, Early Treatment Group*.

b *Relative to the presumed date of infection*.

c *Time from the moment of cART initiation to sample obtaining*.

d *Determinations evaluated at the closest sample obtained before cART initiation*.

e *Versant HIV-1 RNA 3.0 assay, Siemens. Lower and upper detection limits are 50 and 500,000 RNA copies/ml, respectively (1.7 and 5.7 log_10_)*.

f *Determined by flow cytometry*.

g *Determinations evaluated in samples used in this study*.

* *Estimated time to cART initiation, as informed by the clinician*.

Median pre-treatment plasma VL was significantly higher in ET compared to DT (283,441 RNA copies [IQ25-75: 31,443–500,000] and 38,407 RNA copies/ml [IQ 25-75: 11,349–122,260], respectively; *p* = 0.0207). This reflects the fact that DT individuals already reached the VL set point by the moment of cART initiation while ET individuals had not reached that stage yet. All samples obtained after cART initiation and used for this study had undetectable plasma VL (lower limit of detection 50 RNA copies/ml).

Pre-treatment CD4^+^ T-cell counts and CD4/CD8 ratios did not differ between groups (DT: median CD4^+^ T-cell count = 370 cells/μl [IQ25-75: 290.3–450.8], Median CD4/CD8 ratio = 0.45 [IQ25-75: 0.33–0.66]. ET: median CD4^+^ T-cell count = 435 cells/μl [IQ25-75: 334.5–640.5], Median CD4/CD8 ratio = 0.33 [IQ25-75: 0.18–0.68]). Both groups experienced significant improvements in both parameters by 1 year after cART initiation compared to the pre-cART determination (DT: median CD4^+^ T-cell count = 549 cells/μl [IQ25-75: 506.8–732], *p* = 0.002; median CD4/CD8 ratio = 0.755 [IQ25-75: 0.67–1.26], *p* = 0.0005; ET: median CD4^+^ T-cell count = 829 cells/μl [IQ25-75: 625.5–1053], *p* = 0.0005; median CD4/CD8 ratio = 1.32 [IQ25-75: 0.89–1.74], *p* = 0.0002). Moreover, both CD4^+^ T-cell count and CD4/CD8 ratio evaluated on-cART were significantly higher in ET vs. DT (*p* = 0.025 and *p* = 0.014, respectively), mirroring a poorer CD4^+^ T-cell recovery in DT after a longer period prior cART initiation.

### HIV-specific T-cells from DT and ET subjects on cART could be equally expanded *in vitro*

**Figure 2:**
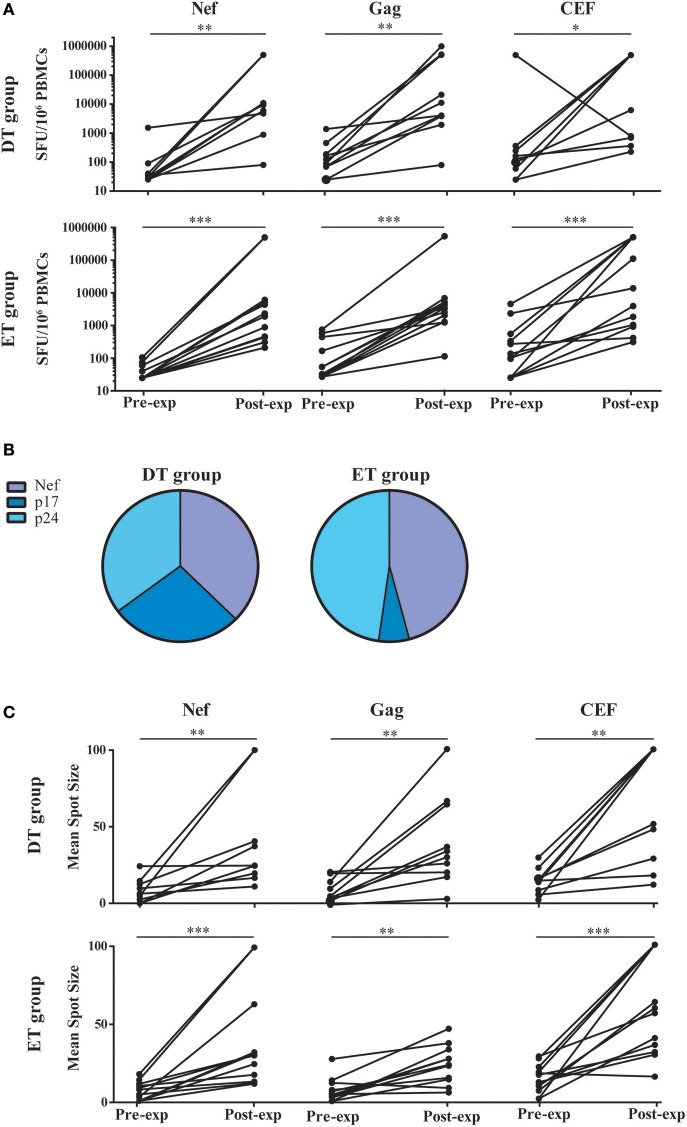
ELISPOT screening of HIV-specific T-cell response before and after *in vitro* expansion, in samples from DT and ET individuals obtained after one year on cART. **(A)** Magnitude of total anti-Nef, anti-Gag (sum of anti-p17 and anti-p24 responses), and anti-CEF IFN-γ-producing cellular responses, expressed as spot forming units (SFU)/10^6^ PBMCs, measured pre- and post-expansion *in vitro* on a subject-by-subject basis (each represented by a line). **(B)** Relative contribution of each antigen to the total HIV post-expansion response expressed as the percentage out of the total sum of the specific response (sum of the magnitude obtained for all Nef, p17 and p24 antigens), for DT (left pie) and ET (right pie) subjects. **(C)** Mean spot size obtained for HIV and CEF peptide pools at pre- and post-expansion conditions. HIV mean spot sizes represent the average mean spot sizes out of all Nef, Gag, and CEF pools for which a positive response was obtained in the ELISPOT assay on a subject-by-subject basis. Intragroup differences were analyzed using Wilcoxon test. Asterisks denote different *P* values: *p < 0.05; **p < 0.01; ***p < 0.005. DT, Delayed treatment (*N* = 10); ET, Early treatment (*N* = 13).

Based on the notion that HIV-specific CD8^+^ T-cell frequency decays significantly following cART initiation, and that the median half-life for the rate of decay is around 40 weeks (16), we first evaluated whether HIV-specific CD8^+^ T-cells from DT and ET subjects after 1 year of cART could be expanded in an *in vitro* model. For this, PBMCs were stimulated with HIV peptide pools spanning Nef and Gag (sum of anti-p24 and p17 responses) proteins or the control CEF pool; plus IL-2 for 14 days and specific responses were evaluated pre- and post-expansion by ELISPOT (Figure 2A). Median pre-expansion responses found in DT were 30 SFU/10^6^ PBMC (IQ25-75 25–65) for Nef, 107.5 (58.75–257.5) for Gag and 147.5 (51.25–366.3) for CEF peptides. No significant differences were found when comparing pre-expansion DT responses with those evaluated in ET, which were as follows: 25 (25–55) for Nef, 40 (25–346.3) for Gag, and 117.5 (25–495) for CEF peptides. After expansion, responses to all antigens tested were significantly increased in both study groups (by 10^3^ times) and were predominantly mediated by CD8^+^ T-cells, as observed later by flow cytometry (as explained below) which is consistent with higher representation of MHC-class I restricted peptides within the PTE peptide pools. Post-expansion magnitude in DT was 9,390 SFU/10^6^ PBMC (IQ25–75 2,800–255,455) for Nef (*p* = 0.0039, compared to the pre-expansion condition), 7,700 (3,415–507,893) for Gag (*p* = 0.002) and 253,155 (617.5–500,000) for CEF (*p* = 0.0176). Similarly, post-expansion magnitude in ET was 2,110 (441.3–5,900) for Nef (*p* = 0.0005), 3,105 (1,365–4,578) for Gag (*p* = 0.0005) and 8,740 (940–500,000) for CEF (*p* = 0.0005). No differences were observed between DT and ET, for any of the antigen used. Then, the relative contribution of each antigen to the total post-expansion anti-HIV response was analyzed to provide an image of the response breadth for both groups (Figure 2B). DT individuals showed an even contribution of anti-Nef, p17 and p24 responses to the total HIV response. Although the distribution was not different from ET (*p* = 0.1786), it is worth highlighting that ET showed a lower contribution of anti-p17 responses and a higher contribution of anti-Nef responses, resulting globally in a narrower response mostly directed toward Nef and p24. Moreover, no differences were found when comparing the pre- and post-expansion distribution intragroups (not shown). Finally, the mean spot size was recorded in both pre- and post-expansion conditions as a measure of the amount of IFN-γ produced by the individual specific T-cells (Figure 2C). Mean spot size significantly increased after expansion with all antigens tested. No significant differences were observed between DT and ET subjects. In sum, the magnitude of HIV-specific cellular responses could be equally expanded *in vitro* both in DT and ET subjects, and cells showed higher potential to secrete IFN-γ after stimulation. Of note, this parameter has been associated with higher T-cell avidity and a polyfunctional profile (13, 48, 49). Finally, DT subjects displayed a broader response to HIV peptide pools after expansion than ET subjects.

### Polyfunctionality of expanded CD8^+^ T-cells is not conditioned by cART initiation timing

**Figure 3:**
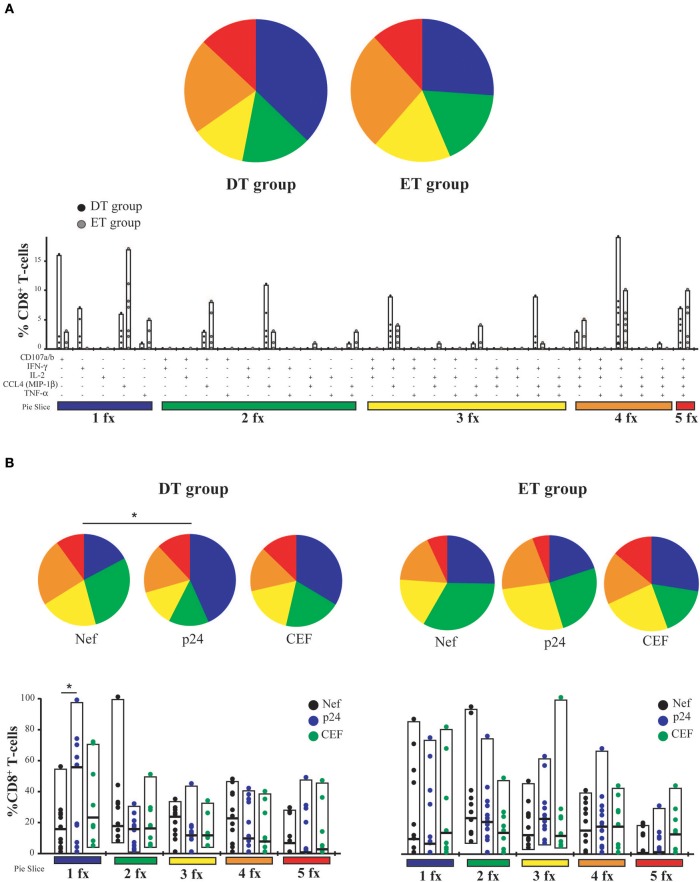
CD8^+^ T-cell polyfunctionality analysis after *in vitro* expansion with specific peptides. **(A)** Pies depict the distribution of mono-(blue), bi-(green), tri-(yellow), tetra-(orange), and penta-functional (red) cells within HIV specific CD8^+^ T-cells, for DT and ET subjects. Bar charts represent the proportion of HIV-specific CD8^+^ T-cells displaying each particular function or combination of functions (31 possible combinations of positive responses). The color code shown at the bottom mirrors the one shown in the pies. Black dots represent DT subjects and gray dots represent ET subjects. Boxes extend from min to max, horizontal bar within boxes represent median values **(B)** Polyfunctional profile for CD8^+^ T-cells with different specificities (Nef, p24 or CEF) within DT group (left panel) and ET group (right panel). This analysis was performed considering cells that were able to mediate either one, two, three, four, or five functions, regardless of any particular function or function combination (block analysis). Analyses were performed with SPICE software. When analyzing global distribution (pies) permutation tests were applied, instead when analyzing bar graphs, Student *T*-test were used. *p < 0.05. DT, Delayed treatment (*N* = 11 for Nef- and p24-specific responses, *N* = 8 for CEF-specific responses); ET, Early treatment (*N* = 12 for Nef- and p24-specific responses, *N* = 9 for CEF-specific responses).

T-cell polyfunctionality has been largely considered an important metric reflecting the quality of the T-cell response (50). Thus, the capacity to degranulate (evaluated by the mobilization of CD107a/b to the plasma membrane) and to produce IFN-γ, IL-2, CCL4 (MIP-1β), and TNF-α was evaluated post-expansion by flow cytometry (gating strategy is shown in Figure S1). From this point forward, only CD8^+^ T-cells expanded with Nef and p24 peptide pools were analyzed. p17-specific cells were excluded because, in this model, they proved limited expansion potential. It was observed that, as aforementioned, CD8^+^ T-cells were preferentially expanded in our model, and CD4^+^ T-cells represented only a small proportion of cells after expansion (data not shown). When analyzing CD8^+^ T-cells expressing each of the functions either alone or in combination, and in consonance with the ELISPOT results, it was first noted that the proportion of responsive cells was much higher than that found when evaluating this parameter directly *ex vivo* (13, 14, 51). This finding is in line with the specific proliferation and survival of HIV-specific cells after the *in vitro* expansion. No significant differences were observed between DT and ET subjects either when polyfunctionality was analyzed as a whole or when functions were analyzed individually or combined (Figure 3A). Then, the same analysis was performed but splitting the responses toward the different HIV antigens and CEF (Figure 3B). In this case, total proportion of mono, bi, tri, tetra, and pentafunctional cells were analyzed independently of any particular function due to the low number of positive responses when fragmenting the analysis. By doing this, no differences were observed in the polyfunctional profile of expanded Nef-specific, p24-specific, even in CEF-specific CD8^+^ T-cells obtained from ET. Only a non-significant reduction in the proportion of pentafunctional Nef-specific and p24-specific cells was observed when compared to CEF-specific cells from the same group. Conversely, expanded p24-specific CD8^+^ T-cells from DT subjects showed an enrichment of monofunctional cells compared to Nef-specific (*p* = 0.042) and CEF-specific (*p* = 0.16) cells. Overall, expanded HIV-specific CD8^+^ T-cells from DT and ET subjects showed a polyfunctional response comparable to CEF-specific responses, with high proportions of tetra and pentafunctional cells.

### Time to cART initiation impacts on CD8^+^ T-cell memory/effector phenotype distribution post-expansion

**Figure 4:**
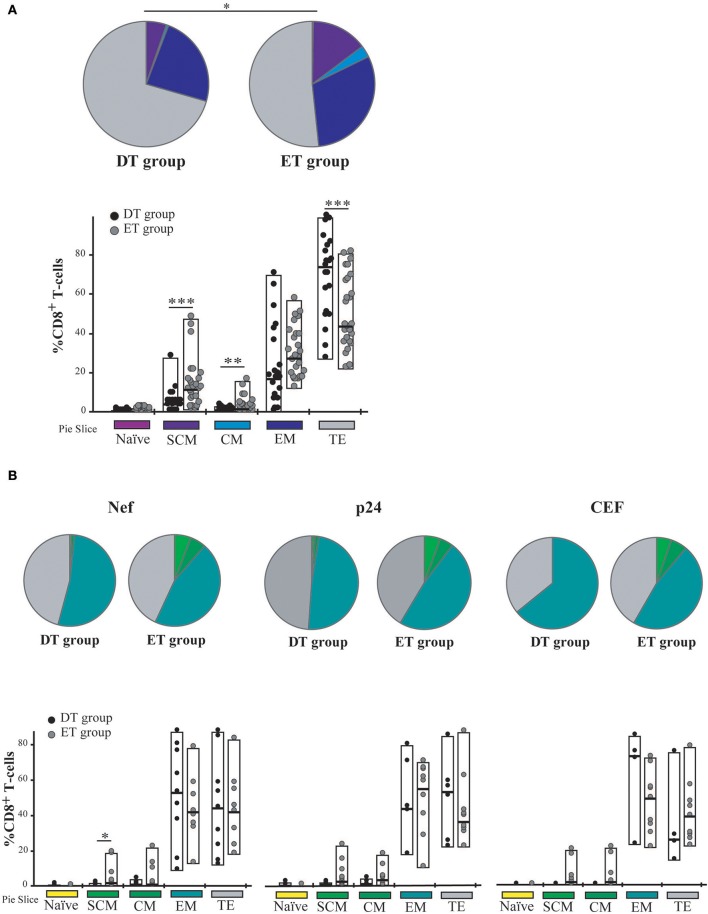
Memory/effector phenotype analysis on expanded CD8^+^ T-cells from DT and ET individuals. **(A)** Distribution of effector/memory subsets in bulk CD8^+^ T-cells analyzed post-expansion. DT, Delayed treatment (*N* = 11 subjects, total 22 responses); ET, Early treatment (*N* = 13 subjects, total 26 responses). **(B)** Distribution of effector/memory subsets in Nef-, p24-, and CEF-specific CD8^+^ T-cells, identified on the bases of cytokine production and/or degranulation capacity. DT, Delayed treatment (*N* = 10 Nef-specific responses, *N* = 8 p24-specific responses, *N* = 5 CEF-specific responses); ET, Early treatment (*N* = 12 Nef-specific responses, *N* = 12 p24-specific responses, *N* = 10 CEF-specific responses). In **(A,B)**, analyses were performed with SPICE software. In bar graphs, the color code shown at the bottom mirrors the one shown in the pies. Black dots represent DT subjects and gray dots represent ET subjects. Boxes extend from min to max, horizontal bar within boxes represent median values. When analyzing global distribution (pies) permutation tests were applied, instead when analyzing bar graphs, Student *T*-test were used. *p ≤ 0.05; **p ≤ 0.01; ***p ≤ 0.005. SCM, Stem cell memory; CM, Central memory; EM, Effector memory; TE, Terminal effector.

Cell phenotype was then evaluated by flow cytometry (Figure S1). Distribution of naïve (T_N_), stem-cell memory (T_SCM_), central memory (T_CM_), effector memory (T_EM_), and terminal effector (T_TE_) T-cells was studied both on bulk and HIV-specific CD8^+^ T-cells. In addition, expression of PD-1 was monitored. As expected, the general memory phenotype distribution was sharply skewed toward an effector phenotype after expansion. When analyzing the distribution of memory phenotype within the bulk CD8^+^ T-cell compartment, the following hierarchy emerged in both groups: T_TE_ >T_EM_ >T_SCM_ >T_CM_ >T_N_ (Figure 4A). However, the global distribution was significantly different between DT and ET (*p* = 0.0051) driven by intergroup differences in the proportion of various sub-populations: ET subjects showed significantly higher proportions of T_SCM_ (median % 11.93 vs. 4.21, *p* = 0.003) and T_CM_ (median % 1.9 vs. 1.00, *p* = 0.016) subsets, and significantly lower proportions of T_TE_ (median % 44.35 vs. 73.70, *p* = 0.003). Percentages of T_N_ cells were minimum in both groups (0.22 and 0.64% in DT and ET, respectively). Within the HIV-specific compartment, T_EM_ and T_TE_ were exclusively found among cells from DT while in ET subjects T_SCM_ and T_CM_ were also observed. The same pattern was observed for Nef-specific and p24-specific responses as well as for the CEF-specific response when analyzed separately (Figure 4B). In this case, the differences observed were not statistically significant, most likely due to the reduced numbers of positive responses when the analysis was segregated across antigens.

**Figure 5:**
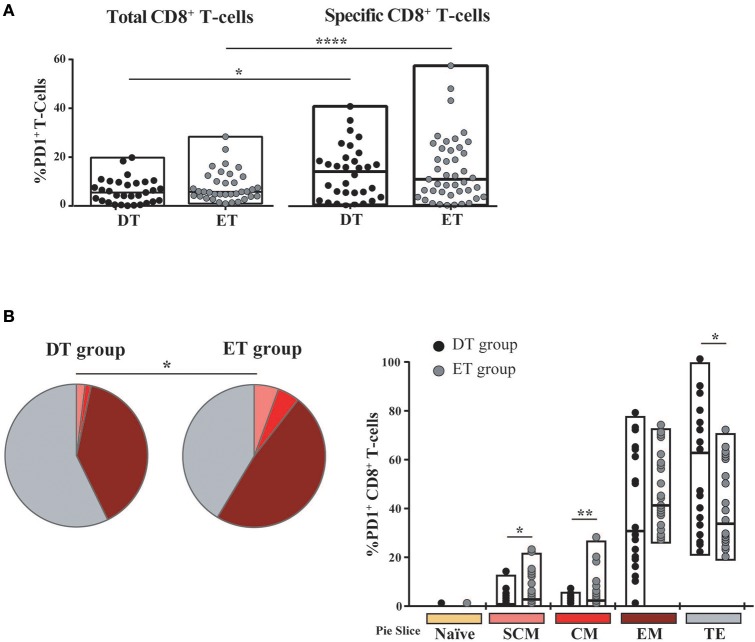
PD-1 expression on expanded CD8^+^ T-cells from DT and ET individuals. **(A)** Post-expansion percentages of bulk and HIV-specific PD-1^+^ CD8^+^ T-cells. Boxes extend from min to max. Horizontal bar within boxes represent the median. *p ≤ 0.05; ****p ≤ 0.0001 according to Wilcoxon's test. **(B)** Distribution of effector/memory phenotypes within bulk PD-1^+^ CD8^+^ T-cells analyzed post-expansion. Analysis was performed with SPICE software. In bar graphs, the color code shown at the bottom mirrors the one shown in the pies. Black dots represent DT subjects and gray dots represent ET subjects. Boxes extend from min to max, horizontal bar within boxes represent median values. When analyzing global distribution (pies) permutation tests were applied, instead when analyzing bar graphs, Student *T*-test were used. *p ≤ 0.05; **p ≤ 0.01. DT, Delayed treatment (*N* = 11 subjects, 21 responses). ET, Early treatment (*N* = 13 subjects, 26 responses). SCM, Stem cell memory; CM, Central memory; EM, Effector memory; TE, Terminal effector.

Then, we sought to analyze PD-1 expression in expanded cells. First, it was observed that the PD-1 expression was markedly lower in expanded cells compared to previous studies in a similar population but measuring PD-1 expression directly *ex vivo* (not shown) (14, 52–54). No significant differences were observed in PD-1 expression either at the bulk or HIV-specific CD8^+^ T-cells compartments, between DT and ET groups (Figure 5A). On the other hand, HIV-specific cells showed significantly higher PD-1 expression compared to the bulk compartment, both in DT (*p* = 0.0268) and ET (*p* < 0.0001) which is in consonance with previous observations in other settings (14, 55, 56). Then, the distribution of different memory/effector phenotypes was investigated within the PD-1^+^ events (only for the bulk compartment) (Figure 5B). The global distribution of subsets was significantly different between groups (*p* = 0.0339), with DT subjects showing an increased representation of CD8^+^ T_TE_ cells within the PD-1^+^ events, compared to ET subjects (*p* = 0.026) who, in turn, showed higher proportions of T_SCM_ and T_CM_ CD8^+^ PD-1^+^ cells (*p* = 0.023 and *p* = 0.013, respectively). Subsequently, we aimed to analyze the PD-1 expression distribution along the different memory/effector phenotypes. Due to the reduced number of events within the T_N_, T_SCM_, and T_CM_ subsets, this analysis could only be performed on the bulk CD8^+^ T_EM_ and T_TE_ subsets. In both DT and ET subjects, PD-1 expression was significantly higher in T_EM_ compared to T_TE_ subsets (Figure S2A). Likewise, T_EM_ CD8^+^ cells showed a significantly higher proportion of degranulating and/or cytokine-producing cells compared to T_TE_ in both groups (Figure S2B) pointing to a rheostat-like function of PD-1 rather than being merely a marker of cell exhaustion.

Overall, both at the bulk and HIV-specific compartments, ET subjects showed a preservation of early differentiated cells (T_SCM_ and T_CM_), which was also represented as a higher proportion of T_SCM_ and T_CM_ cells within the PD-1^+^ compartment. DT subjects showed a differentiated profile with increased representation of cells with effector phenotype (T_EM_ and T_TE_). PD-1 expression was concentrated in HIV-specific CD8^+^ T_EM_ cells.

### Expanded CD8^+^ T-cells from DT and ET subjects were highly capable of mediating antiviral activity

**Figure 6:**
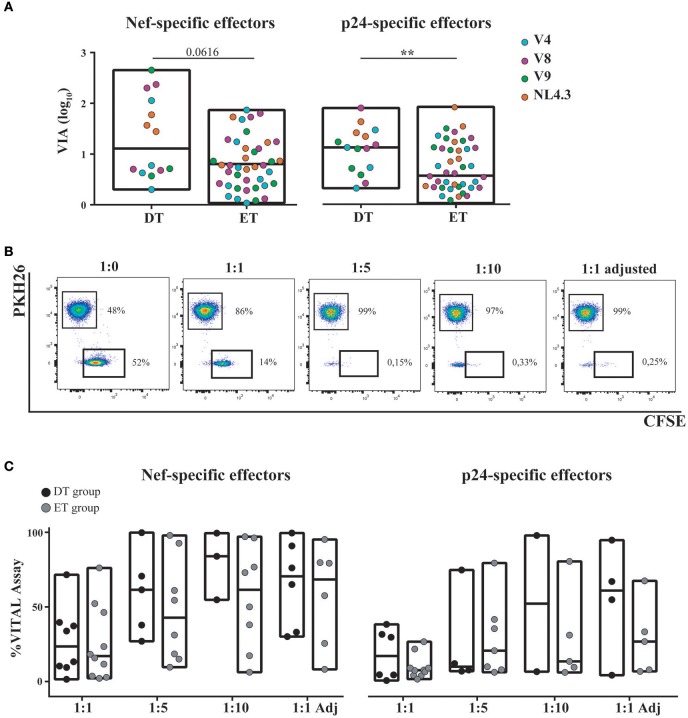
Capacity of *in vitro* expanded CD8^+^ T-cells to exert viral inhibition by cytolytic and non-cytolytic mechanisms. **(A)** *In vitro* viral inhibition assay (VIA) mediated by Nef-specific (left panel) and p24-specific (right panel) CD8^+^ T-cells. VIA was expressed as the log_10_ of the proportion of p24 antigen lost when effector CD8^+^ T-cells were present in the culture, compared to target cells (infected autologous CD4^+^ T-cells) alone. Dots represent individual subjects. The different viral strains employed in the experiment are displayed in a different color. DT, Delayed treatment (*N* = 4 subjects, 14 Nef-specific responses and 15 p24-specific responses); ET, Early treatment (*N* = 12 subjects, 40 Nef-specific responses and 41 p24-specific responses). **(B)** Dot-plots obtained in one representative VITAL assay. At the 1:0 T:E ratio (far left panel), an even distribution of peptide-loaded (CFSE^+^PKH26^−^) targets and not-loaded targets (CFSE^−^PKH26^+^) targets is observed. From left to right, increasing ratios of effectors (1:1, 1:1 adjusted [i.e., 1:1 ratio adjusted to proportion of specific CD8^+^ T-cell effectors as determined by intracellular staining and flow cytometry], 1:5 and 1:10) are shown and the concomitant loss of antigen-loaded targets is observed. **(C)** Percentage of specific lysis exerted by Nef-specific (left panel) and p24-specific (right panel) CD8^+^ T-cells, as determined by the VITAL assay. Results for DT and ET subjects are shown at the different T:E ratios evaluated. DT, Delayed treatment (*N* = 7 subjects for Nef-specific responses and *N* = 6 subjects for p24-specific responses). ET, Early treatment (*N* = 10 subjects, both for Nef- and p24-specific responses). Black dots represent DT subjects and gray dots represent ET subjects. In **(A,C)**, boxes extend from min to max values. Horizontal lines within boxes represent the median values. In **(A)**, intergroup differences were analyzed using Mann-Whitney test; **p < 0.01.

The capacity of *in vitro* expanded CD8^+^ T-cells to exert viral inhibition by cytolitic and non-cytolitic mechanisms was evaluated by two different assays (see M&M and Figure 1). First, the VIA assay (which measures the magnitude of the overall CD8^+^ T-cell antiviral potency comprising both cytolytic and non-cytolytic pathways) (39) was performed to evaluate the ability of expanded cells (either expanded with Nef or p24 peptides) to suppress the replication of one lab-adapted HIV strain (NL4-3) and three T/F primary strains (V4, V8, and V9). A significant capacity to mediate viral inhibition was observed both for Nef-specific and p24-specific cells from DT and ET subjects toward all viruses assayed (Figure 6A). Within Nef-specific CD8^+^ T-cells, no significant differences were found between groups in their capacity to mediate VIA, although a trend was evident toward DT individuals displaying a higher capacity (*p* = 0.0616). In contrast, p24-specific CD8^+^ T-cells from DT subjects showed a significantly stronger antiviral activity compared to p24-specific cells from ET subjects (*p* = 0.005). No significant differences were observed when analyzing Nef-specific and p24-specific cells within each study group. In parallel, the VITAL assay (which depicts direct cytotoxicity) was performed. Figure 6B shows dot-plots obtained from one representative assay, which depicts specific progressive loss of antigen-loaded CFSE^+^ target cells as effectors are added at increasing ratios. Compiled data indicated that no significant difference could be observed between DT and ET individuals at any E:T ratio evaluated, regardless of CD8^+^ T-cell specificity (Figure 6C). When performing an intragroup response analysis (i.e., comparing Nef-specific vs. p24-specific CD8^+^ T-cells from DT or ET subjects), no differences were found (Wilcoxon's test *p* > 0.05). Only a tendency toward Nef-specific CD8^+^ T-cells was evidenced showing an improved capacity to mediate a direct cytolytic activity compared to p24-specific CD8^+^ T-cells in both groups.

### Full differentiation, polyfunctionality, and PD-1 expression of CD8^+^ T-cells relate to antiviral function in this expanded model

**Figure 7:**
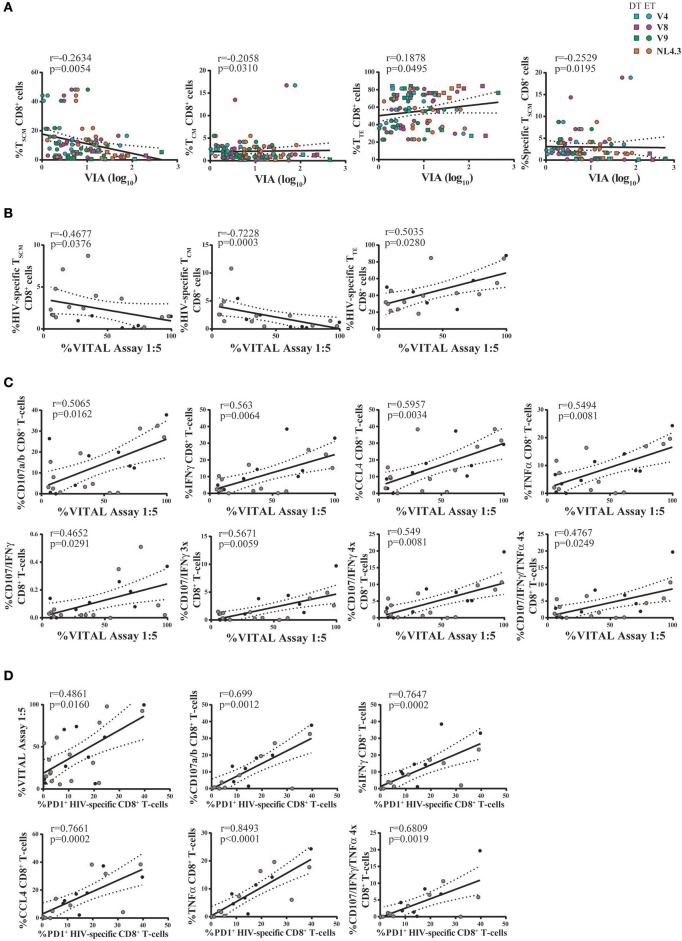
Correlation analyses between antiviral activity versus cell phenotype and functionality of expanded CD8^+^ T-cells. **(A)** Correlations between VIA magnitude (evaluated against all viruses and merging data from Nef-specific and p24-specific effectors) and bulk and HIV-specific CD8^+^ T-cell memory phenotype. Square dots represent DT subjects and round dots represent ET subjects. The different viral strains employed in the VIA experiment are displayed in a different color, matching the color code showed in Figure 6A. **(B,C)** Correlations between VITAL magnitude and the proportions of HIV-specific CD8^+^ T-cell memory phenotype **(B)**, and the proportions of CD8^+^ T-cell functionality **(C)**. **(D)** Correlations between the percentage of PD-1 expressing HIV-specific CD8^+^ T-cells and cell functionality. **(B–D)** Black dots represent DT subjects and gray dots represent ET subjects. *r* and *p* values correspond to Spearman's correlations.

Thus, we sought to investigate the relationship between CD8^+^ T-cell phenotype and functionality (in terms of cytokine production and degranulation capacity) post *in vitro* expansion, and the magnitude of antiviral activity measured by VIA and VITAL assays. For this, results obtained with Nef- and p24-specific effectors were merged, due to no intragroup differences (i.e., within DT or ET subjects). First, a potential association with the results from the VIA assay was investigated. Activity against all viruses was examined. Correlation analysis revealed that the magnitude of VIA was inversely correlated with the proportion of bulk CD8^+^ T_SCM_ cells (*r* = −0.2634, *p* = 0.0054), bulk CD8^+^ T_CM_ cells (*r* = −0.2058, *p* = 0.031), and directly with the proportion of bulk CD8^+^ T_TE_ cells (*r* = 0.1878, *p* = 0.0495). All correlations remained statistically significant after adjusting for multiple comparisons (*p* = 0.0270, *p* = 0.0775, and *p* = 0.0825, respectively). Furthermore, an inverse correlation with the proportion of HIV-specific CD8^+^ T_SCM_ cells was found (*r* = −0.2529, *p* = 0.0195, adjusted *p* = 0.0713; Figure 7A). On the other hand, no significant correlations were found when analyzing the capacity of cells to mediate degranulation and/or secrete cytokines. Second, the magnitude of VITAL assay, at 1:5 T:E ratio, was inversely correlated with the proportion of HIV-specific CD8^+^ T_SCM_ cells (*r* = −0.4677, *p* = 0.0376, adjusted *p* = 0.0627), inversely with HIV-specific CD8^+^ T_CM_ cells (*r* = −0.7228, *p* = 0.0003, adjusted *p* = 0.015), and directly correlated with the proportion of HIV-specific CD8^+^ T_TE_ cells (*r* = 0.5035, *p* = 0.0280, adjusted *p* = 0.0627) (Figure 7B). Additionally, correlations between VITAL magnitude and CD8^+^ T-cell functionality were also found: direct correlations with the proportion of degranulating cells (*r* = 0.5065, *p* = 0.0162, adjusted *p* = 0.0432), IFN-γ-producing (*r* = 0.563, *p* = 0.0064, adjusted *p* = 0.0259), CCL4-producing (*r* = 0.5957, *p* = 0.0034, adjusted *p* = 0.0259), and TNF-α-producing CD8^+^ T-cells (*r* = 0.5494, *p* = 0.0081, adjusted *p* = 0.0259), as well as with proportions of polyfunctional cells able to degranulate and secrete IFN-γ alone or plus one or two extra functions, regardless of any particular one (*r* = 0.4652, *p* = 0.0291, adjusted *p* = 0.0582; *r* = 0.5671, *p* = 0.0059, adjusted *p* = 0.0259, *r* = 0.549, *p* = 0.0081, adjusted *p* = 0.0259, respectively), and also tetrafunctional cells able to degranulate, express IFN-γ and TNF-α plus 1 extra functions (*r* = 0.4767, *p* = 0.0249, adjusted *p* = 0.0569) (Figure 7C). Finally, PD-1 expression within HIV-specific cells proved to directly correlate with direct cytotoxicity (VITAL assay 1:5 ratio; *r* = 0.4861, *p* = 0.0160, adjusted *p* = 0.032), the proportion of degranulating cells (*r* = 0.699, *p* = 0.0012, adjusted *p* = 0.0063), IFN-γ-producing CD8^+^ T-cells (*r* = 0.7647, *p* = 0.0002, adjusted *p* = 0.0014), CCL4-producing CD8^+^ T-cells (*r* = 0.7661, *p* = 0.0002, adjusted *p* = 0.0014) and TNF-α producing CD8^+^ T-cells (*r* = 0.8493, *p* < 0.0001, adjusted *p* = 0.0014). Interestingly, PD-1 expression within HIV-specific cells also directly correlated with the proportion of degranulating polyfunctional cells that concomitantly secrete IFN-γ, TNF-α plus one extra function (*r* = 0.6809, *p* = 0.0019, adjusted *p* = 0.008) (Figure 7D). Overall, this data points toward the need to fully differentiate into an effector phenotype to gain maximum antiviral activity in both assays. VITAL assay better correlated with subsets of functions containing degranulating cells, thus reflecting the nature of the assay. Of note, correlations with the proportion of IFN-γ and TNF-α-producing CD8^+^ T-cells might be driven by a high coexpression with CD107a/b^+^ cells. Interestingly, PD-1 appeared as a marker of polyfunctional expanded cells able to mediate direct cytolysis.

## Discussion

Combination antiretroviral treatment has largely improved HIV^+^ individual's quality of life and expectancy. Nevertheless, it cannot eliminate the viral reservoir by itself, precluding thus the cure of the infection. Many strategies have been proposed to eradicate the viral reservoir. The success of some of these strategies, such as the “shock and kill” approach, will rely mainly on the ability of HIV-specific CD8^+^ T-cells to clear reactivated infected cells. Nevertheless, our knowledge regarding the quality of remaining CD8^+^ T-cell responses on-cART is not accurate. Pivotal studies indicated that cART-treated individuals retain CD8^+^ T-cells that, upon *ex vivo* expansion, are able to exert antiviral activity *in vitro*, even against reactivated cells (i.e., latently infected CD4^+^ T-cells exposed to latency reversal agents) (57–59), although the depletion of infected cells is not complete (60). Yet, the functional and phenotypic characteristics needed to boost in the quest of a functional cure are not thoroughly understood. Here, we performed a comprehensive evaluation of the quality of *in vitro* expanded CD8^+^ T-cells obtained from HIV^+^ subjects who initiated cART at early (<4 month) or delayed (>4 month) time-points post-infection. Results indicated that (i) Nef- and p24-specific T-cell responses could be expanded *in vitro*, both in DT and ET subjects; (ii) expanded cells were highly polyfunctional; (iii) after expansion, the memory/effector differentiation profile was sharply skewed toward an effector phenotype, although ET subjects showed preserved proportions of T_N_, T_SCM_, and T_CM_ subsets; (iv) expanded CD8^+^ T-cells were highly capable of mediating antiviral activity and the magnitude of these activities were related to the proportion of fully-differentiated effector cells as well as with CD8^+^ T-cell functionality; (v) PD-1 expression in expanded cells was mainly concentrated in HIV-specific T_EM_ CD8^+^ cells and correlated to cell polyfunctionality and cytolytic activity.

Compiling evidence depicts the profound benefits that early initiation of cART has on both virologic and immunologic parameters in infected individuals (26, 27, 31, 47). Longer antigen persistence was shown to be deleterious on HIV-specific CD8^+^ T-cells, resulting in an exhausted and hyperactivated immune response (27, 61). Furthermore, the latent reservoir is not only larger in size but also altered in its composition, possibly containing variants resistant to dominant CD8^+^ T-cell responses, rendering immune eradication therapies less efficient in individuals initiating cART during chronic infection (58). So far, few works have studied the differences in breadth and magnitude of CD8^+^ T-cell responses arising from starting cART at different time-points after infection (54, 62, 63), but no evaluation has been focused on how those differences impact on the final antiviral activity, an important issue to be addressed when assessing functional cure strategies. In this context, we aimed to assess whether the moment of cART initiation (relative to the presumed date of infection) has an impact on post-expansion HIV-specific CD8^+^ T-cell responses.

Because CD8^+^ T-cell responses in subjects on cART are dramatically diminished (15–20), an *in vitro* cell expansion protocol was implemented. Several and diverse works have proven its utility in boosting HIV-specific responses to optimal activity (23, 40, 54, 57–59, 64–67). This work analyzed HIV-specific CD8^+^ T-cell responses against Gag and Nef peptide pools based on a number of reasons: (1) during acute infection, Nef is the immunodominant target (68–70), though the response broadens afterwards to epitopes within other viral proteins including Gag (71–75), (2) higher frequencies of CD8^+^ T-cells with polyfunctional responses to Gag and Nef were associated with lower viral set-points (76), (3) Gag-specific CD8^+^ T-cell responses were associated with virus control in different settings (72–74, 77); particularly, early anti-Gag immunodominance was associated with improved antiviral activity and also with lower rate of disease progression (13); (4) expandable Gag responses were associated with strong antiviral activity and lower residual viral loads in Elite Controllers (40, 66); (5) Nef protein is detectable in PBMCs from subjects under cART (78) and (6) CD4^+^ T-cells from individuals on prolonged therapy contain “defective” proviral sequences that are able to express Nef (79). Despite being the CD8^+^ T-cell response very low and at times almost undetectable before stimulation, we were able to specifically and significantly expand it in both groups of individuals. However, DT subjects displayed a more homogeneous response, in terms of specificity, than ET subjects, for whom the post-expansion response was narrower. This could likely occur due to a longer period of antigen stimulation, which would have allowed CD8^+^ T-cells from DT to develop into a broader-spectrum of specificities to different viral antigens. In consonance with the results obtained by Deng et al. a broader CD8^+^ T-cell response would be favorable when the elimination of reactivated viruses is required (58). Nevertheless, it is worth noting that anti-Nef and anti-p24 responses were equally expandable in both groups.

Early reports comparing the capacity of CD8^+^ T-cells to degranulate and secrete multiple soluble mediators upon stimulation, between individuals with progressive vs. long-term nonprogressive HIV infection, have suggested that CD8^+^ T-cell polyfunctionality would be a functional correlate of virus control (76, 80). Later, it was shown that polyfunctionality does not necessarily correlate with disease progression (13). Instead, lack of T-cell polyfunctionality would be the consequence of constant antigen stimulation during viremic chronic infection, which ultimately may lead to cell exhaustion and functional impairment (81, 82). In any case, CD8^+^ T-cell polyfunctionality relates to a beneficial response. Here, a significant proportion of peptide-expanded CD8^+^ T-cells presented the ability to mediate different and simultaneous functions. In this line, several reports have evidenced the significant presence of polyfunctional cells upon expansion with specific peptides (40, 48). Particularly, Ndhlovu et al. (40) showed that, after expansion with a similar protocol to the one used here, Elite controllers had significantly higher frequencies of polyfunctional cells compared to a group of chronically infected subjects on cART. Contrary to the findings of Ndhlovu et al. we did not observe differences between study groups, i.e., polyfunctionality was equally evident in DT and ET subjects. One point to be considered is that DT and ET groups are less polar than Elite controllers and chronics on cART. Thus, more subtle differences might exist between DT and ET which could not be evidenced here. Regarding time to cART initiation, a similar study also indicated that it did not impact CD8^+^ T-cell polyfunctionality profile, although evaluated directly *ex vivo* (54).

CD8^+^ T-cells follow a progressive pathway of differentiation from naïve T-cells into different memory/effector subsets. Each subset is characterized by self-renewal, proliferative and functional attributes (83). Memory CD8^+^ T-cell differentiation in progressive HIV infection is severely skewed, reflecting improper terminal differentiation of effector cells (14, 84, 85). Interestingly, the early deterioration of CD8^+^ T-cell differentiation pathway was directly associated with disease progression (14, 86), while effective cART seems not to restore this phenomenon completely (65). Here, CD8^+^ T-cell memory/effector differentiation was evaluated post-expansion in cells from DT and ET subjects. In this condition of strong antigen triggering and cytokine-driven proliferation signaling, a considerable extent of differentiation to effector phenotypes was expected. However, this was not equally represented by the two study groups included in this work: ET subjects had increased proportions of T_N_, T_SCM_, and T_CM_ upon expansion. Initially, this could have been considered as an advantage since less differentiated cells with improved proliferative and self-renewal capacities were preserved. However, as will be later discussed, it inversely correlated with antiviral function. CD8^+^ T_SCM_ cells have been described as a subset of antigen-experienced cells that retain a core of genes expressed by T_N_ cells, and also display superior ability to proliferate homeostatically and persist in the long term (83). Recently, it has been reported that HIV^+^ subjects receiving cART have higher proportions of CD8^+^ T_SCM_ cells and that this proportion even increases as time on cART extends (87). Since DT and ET subjects have a similar median time on cART at the moment of sampling, it would be interesting to evaluate if a higher representation of CD8^+^ T_SCM_ cells after expansion could be reflecting an increase in the frequency of T_SCM_ cells before expansion. This in turn, would be a consequence of the earliest cART initiation. For regimens of adoptive cell transfer, widely explored in the field of cancer and Epstein-Barr virus–associated malignancies, it is known that CD8^+^ T-cell differentiation widely affects the anti-tumoral response: although effector memory cells have fully functional differentiation, the diminished proliferative capacity severely compromises the success of the adoptive transfer because the effect is transient. Thus, strategies for limiting *ex vivo* differentiation but retaining functional differentiation are being evaluated (88). The same reasoning could apply in this context: ET subjects harbor the advantage of a preserved T_SCM_ subset but strategies to potentiate their antiviral activity should be explored.

Apart from memory/effector phenotype, the expression of PD-1 was investigated post-expansion. PD-1 is an inhibitory coreceptor that is involved in the fine-tuned regulation of the threshold of antigen responses of T and B cells. It is endowed with unique modulatory functions where its presence/absence matters but also its expression level is crucial (89, 90). During chronic viral infections, extremely high and persistent PD-1 expression has been found in T cells. This PD-1^high^ phenotype has been consistently associated with a state of cellular exhaustion in the context of different persistent viral infections, including HIV (14, 55, 56, 89, 91, 92). In this study, PD-1 expression in expanded CD8^+^ T-cells was low compared to other reports measuring PD-1 expression directly *ex vivo*, as already mentioned. This could be the result of PD-1 down-modulation or death of PD-1^+^ cells during expansion. The latter would be in consonance with the notion that exhausted PD-1^+^ cells show decreased telomerase activity and telomere lengths rendering them more susceptible to cell death (93, 94). Expanded PD-1^+^ cells were concentrated in the HIV-specific compartment, particularly within CD8^+^ T_EM_ and T_TE_ cells. In addition, positive correlations were found between PD-1 expression on expanded HIV-specific cells and CD8^+^ T-cell functionality. This is in line with data provided by Hokey et al., who found high PD-1 expression on cells with activated memory and effector phenotypes despite decreased telomere lengths, suggesting PD-1 could play a costimulatory role in CD8^+^ T-cell populations (95). Moreover, a recent report showed that tumor-reactive CD8^+^ T-cells that persist after adoptive cell-transfer therapy, and associate with tumor regression, are mostly polyfunctional and simultaneously express high levels of PD-1 (96). This data highlights the fact that the PD-1 role in the context of viral reactivation needs further research. Many studies have already proven the benefits of PD-1 signaling inhibition by anti-PD-1L (anti-PD-1 ligand] at the moment of CD8^+^ T cell stimulation, (93, 97). Combination therapies that stimulate and simultaneously modulate the specific immune response (through PD-1 blocking for instance) are believed to be the optimal approaches for future therapeutic schemes (98, 99).

Then, we aimed to analyze whether CD8^+^ T-cells quality and phenotype would have an impact on the global capacity to mediate viral inhibition. To attain this, two different approaches were used. VIA is a versatile technique that allowed us to investigate CD8^+^ T-cell-mediated inhibition of viral replication of four different virus strains, three HIV-1 primary isolates and a laboratory strain. Even though it provides a global picture of the antiviral response, it cannot discriminate if cytotoxicity plays a relevant antiviral role in this expansion model. To assess this aspect, Herman's VITAL assay was implemented. This method particularly evaluates the direct cytotoxicity, requiring cell-to-cell contact between peptide-loaded target cells and effectors. Thus, this was carried out concomitantly with VIA to assess the contribution of cytolytic mechanisms to the global antiviral response. We considered relevant to perform such a discrimination because in a context of functional cure, strong cytolytic activity would be preferred over non-cytolytic mechanisms to eliminate reactivated cells (100). In this fashion, these experiments provide complementary data, granting a larger picture of the immune response. Here, both Nef- and p24-specific cells from DT and ET individuals were able to mediate VIA against the broad repertoire of viruses assayed, including clinically relevant viral strains. HIV-specific cells from DT group were prone to display greater inhibitory activity than ET but it was only statistically different from p24-specific cells. Bulk CD8^+^ T-cell differentiation level correlated with VIA magnitude, stating that complete differentiation was needed to be achieved in order to mediate viral inhibition. We have already proven that terminal memory differentiation was needed to exert optimal antiviral activity in subjects off-cART and measured directly *ex vivo* (14). Here, this association was maintained even after expansion and in subjects on cART. This is consistent with reports that proved effector memory T-cells efficiency at controlling new infections because they are endowed with more immediate effector functions (27, 101). For VITAL assay, no differences were found between groups. Furthermore, VITAL magnitude correlated with a higher proportion of HIV-specific terminal effector cells. We also found that cells able to degranulate and secrete IFNγ or TNFα, either as unique functions or combined, exerted a greater cytolityc activity. In this line, it has been shown that CD107a/b surface expression was associated with the capacity of antigen-specific CD8^+^ T-cells to eliminate infected cells as observed in VIA assays (13, 102). Additionally, CCL4 has been shown to interfere with HIV infection by blocking the binding to CCR5 coreceptor and has been associated with higher VIA magnitude (102). In our model, no differences were found in the total percentage of CCL4-expressing CD8^+^ T-cells between groups, which is consistent with the lack of difference in VIA magnitude. To date, no reports associated VITAL assays with the level of degranulation post-expansion in HIV-specific cells, representing a novel finding. Of note, most correlations found for VITAL assay were driven by results obtained for Nef-specific responses. Moreover, a tendency toward Nef-specific cells able to mediate stronger direct cytotoxicity was evidenced. Given that CD4^+^ T-cells from HIV^+^ subjects on treatment upregulate HIV-1 mRNA within 1 h of stimulation and produce extracellular virus as early as six-h poststimulation, CD8^+^ T-cells that recognize specifically early gene products, such as Nef, would be most beneficial in functional cure approaches. Additionally, they would effectively kill reactivated cells before viral spread and Nef-mediated MHC-downmodulation (67). Indeed, recent findings indicated that ongoing Nef-expression in treated subjects is associated with the maintenance of Nef-specific CD8^+^ T-cells (28). Not only our results suggest that direct cytotoxicity was mediated more efficiently by Nef-specific CD8^+^ T-cells, but also, for the reasons above stated, it would be an important target to boost in functional cure approaches and vaccine formulations.

Overall, this is a novel comprehensive study evaluating the quality of *in vitro* expanded CD8^+^ T-cells from DT and ET HIV^+^ subjects with relevant implication in the design and evaluation of functional cure strategies relying on CD8^+^ T-cell-mediated killing among others. Limitations of this study include, but might not be restricted to: (i) the use of a strong and prolonged stimulation protocol for T-cell expansion might mask subtle inter-group differences, for instance as a consequence of activation-induced cell death of certain cell subsets and survival of others common to both groups, thus we cannot exclude the possibility that with alternative expansion protocols (such as by using IL-7 or IL-15) differences may arise; (ii) identification of HIV-specific cells by combining molecules with the same fluorochrome leads to the underestimation of the specific response which might be mistakenly considered within the bulk compartment; (iii) production of perforins and granzymes, which were already described as key components of CD8^+^ T-cell antiviral function, were not measured, and thus not accounted, for depicting CD8^+^ T-cell quality; (iv) DT group is highly heterogenous which might potentially lead to data misinterpretation; (v) ET subjects might have initiated treatment even after seroconversion so they do not represent really early treated subjects such as those defined in other cohorts (27). This last point might instead be interpreted as an advantage in terms of potential translation of the results to the every-day practice. Detection of an acutely infected subject is extremely unusual, being mostly diagnosed at late Fiebig stages (IV to VI). Therefore, the scenario presented here would better approximate to a real context.

In sum, low frequency of HIV-specific CD8^+^ T-cells from subjects on-ART for at least 1 year were readily expandable *in vitro* and retained antiviral functions. Minor qualitative differences were observed in expandable CD8^+^ T-cell responses between DT and ET subjects, mostly related to the memory/effector differentiation profile. Thus, the initiation of cART before the viral set-point did not represent a major benefit in terms of post-expansion CD8^+^ T-cell quality in our model. In other words, having started cART during chronic infection would not represent an obstacle to undergo immunomodulatory approaches. Studies aimed at determining how the quality of the pre-cART response relates to the post-expansion scenario and how all these parameters relate to the viral reservoir are being conducted. Although we showed here that *in vitro* expansion results in a fully antiviral functional response, it remains to be elucidated if this kind of responses (with the proper specificity and functionality), can be elicited *in vivo* (through therapeutic vaccination, for instance) or if an adoptive transfer strategy would be the best option to achieve a functional cure for HIV. Additionally, the inclusion of immunomodulators (immune checkpoint inhibitors and/or agonist of stimulatory pathways) should be considered to overcome the intrinsic immune damage caused by the infection in each particular subjects, leading to the development of tailor-made regimens. In any case, these results add important information to rationally design and/or evaluate future strategies to modulate not only anti-HIV T-cell responses but also immune responses toward other persistent pathogens.

## Author contributions

GT conceived the study. JS, YG, and GT designed the experiments. JS, MR, CT, MC, and YG performed experiments. MF, OS, and NL contributed samples and gathered clinical data. JS, MG, HS, NL, YG, and GT analyzed and interpreted the data. JS and GT wrote the manuscript. All authors read and approved the final version.

### Conflict of interest statement

The authors declare that the research was conducted in the absence of any commercial or financial relationships that could be construed as a potential conflict of interest.

## Supplementary material

Figure S1 Gating strategy used for the identification of the studied cellular populations, by flow cytometry. **(A)** To study CD8^+^ T-cell polyfunctionality, initial gating was performed on a forward scatter area (FSC-A) vs. FSC-height (FSC-H) plot to remove doublets. Dead cells were then excluded on the bases of Zombie NIR fluorescence. Then, gating was performed on CD14/CD19 negative cells in order to exclude monocytes and B lymphocytes, and then small lymphocytes were selected in FSC vs. side scatter (SSC) plot. Subsequently, CD3^+^ cells were gated in a CD3 vs. SSH dot plot, and then a CD8 vs. CD4 dot-plot was constructed to identify CD8^+^ events. To study CD8^+^ T-cell polyfunctionality, plots were derived from the CD8^+^ gate to study each particular function: degranulation (evidenced as CD107a/b mobilization) and production of IFN-γ, IL-2, CCL4, and TNF-α. Cells capable of exerting multiple functions simultaneously (degranulating and/or secreting multiple cytokines; 2, 3, 4, or 5 functions) were identified using the Boolean gating strategy available at FlowJo v10 software. **(B)** For the phenotype panel, the initial gating strategy was identical to the polyfunctionality panel up-to-the point of CD8 vs. CD4 plot. There, CD8^+^ events were gated to define bulk CD8^+^ T-cells and a CD8 vs. FITC plot was derived to identify HIV-specific CD8^+^ T-cells (defined as the ones degranulating and/or expressing cytokines, all stained in FITC). Subsequent analyses were performed on both populations as shown by overlaid dot-plots and overlaid histograms. To analyze the distribution of the different phenotype subsets, CD45RO vs. CCR7 density plots were constructed to identify central memory T-cells (T_CM_, CCR7^+^/CD45RO^+^), effector memory T-cells (T_EM_, CCR7^−^/CD45RO^+^) and terminal effector T-cells (T_TE_, CCR7^−^/CD45RO^−^). CD95 expression was analyzed within the CD45RO^−^CCR7^+^ cells thus defining naïve T-cells (T_N_, CCR7^+^/CD45RO^−^/CD95^−^) and stem-cell memory T-cells (T_SCM_, CCR7^+^/CD45RO^−^/CD95^+^). Additionally, PD-1 expression was evaluated. In **(A,B)** illustration data represent cells derived from one representative subject, stimulated for 14 days with the HIV Nef peptide pool.

Figure S2 **(A)** Proportion of PD-1^+^ cells observed post-expansion on bulk CD8^+^ TEM and TTE cells from DT and ET individuals. **(B)** Proportion of HIV-specific cells (either Nef-specific or p24-specific) cells, identified on the bases of cytokine production and/or degranulation capacity, observed post-expansion on CD8^+^ TEM and TTE cells from DT and ET individuals. In **(A,B)**, boxes extend from min to max. Horizontal bar within boxes represent the median. ^****^*p* ≤ 0.0001 according to Wilcoxon's test.
