# Supplementary figures and images for: Phenotype, Polyfunctionality, and Antiviral Activity of in vitro Stimulated CD8+ T-Cells From HIV+ Subjects Who Initiated cART at Different Time-Points After Acute Infection
Source: Front Immunol. 2018 Oct 23;9:2443. doi: 10.3389/fimmu.2018.02443 (PMC6205955; doi:10.3389/fimmu.2018.02443)

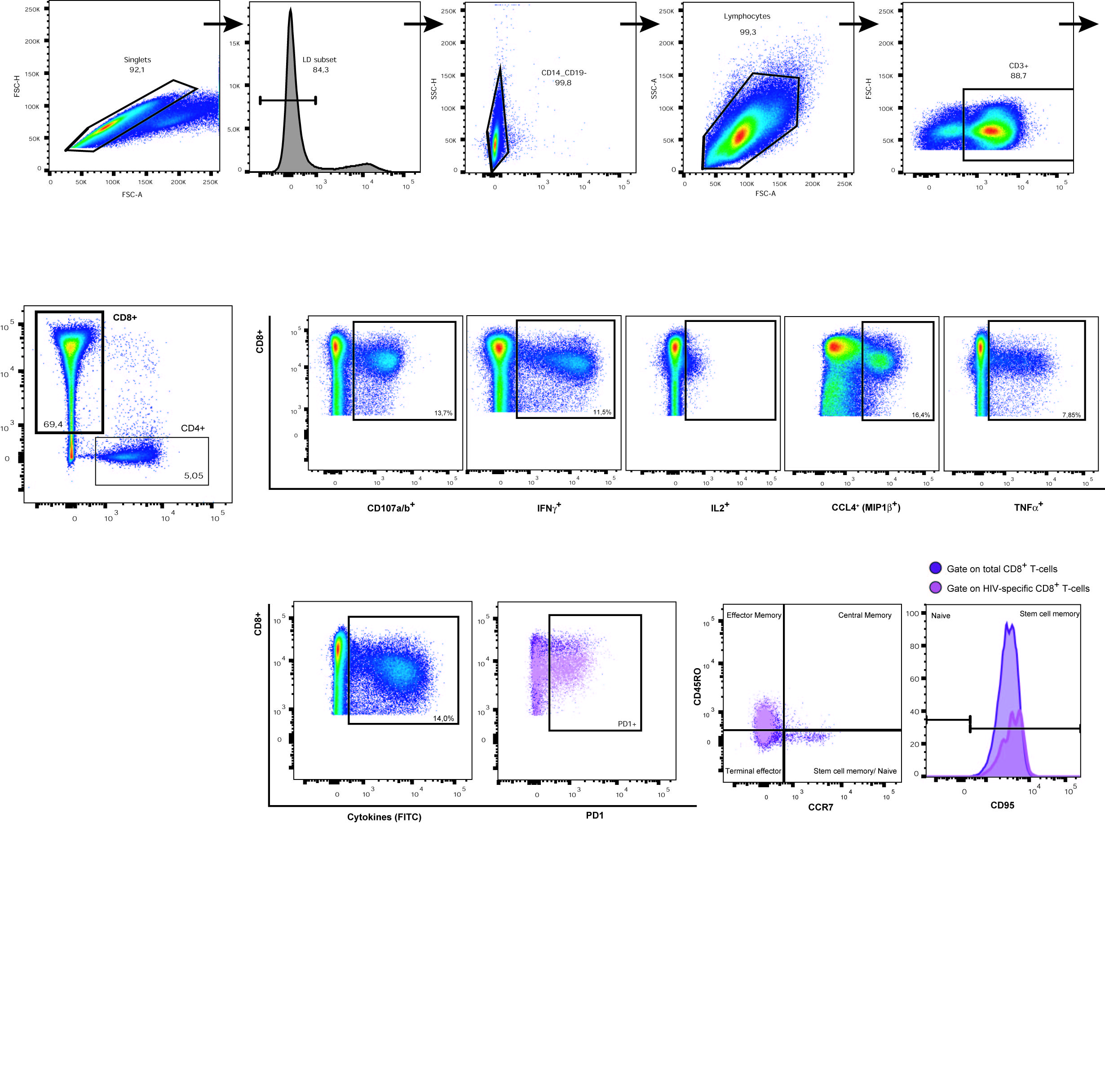

Supplement: Figure S1 — Gating strategy used for the identification of the studied cellular populations, by flow cytometry. (A) To study CD8+ T-cell polyfunctionality, initial gating was performed on a forward scatter area (FSC-A) vs. FSC-height (FSC-H) plot to remove doublets. Dead cells were then excluded on the bases of Zombie NIR fluorescence. Then, gating was performed on CD14/CD19 negative cells in order to exclude monocytes and B lymphocytes, and then small lymphocytes were selected in FSC vs. side scatter (SSC) plot. Subsequently, CD3+ cells were gated in a CD3 vs. SSH dot plot, and then a CD8 vs. CD4 dot-plot was constructed to identify CD8+ events. To study CD8+ T-cell polyfunctionality, plots were derived from the CD8+ gate to study each particular function: degranulation (evidenced as CD107a/b mobilization) and production of IFN-γ, IL-2, CCL4, and TNF-α. Cells capable of exerting multiple functions simultaneously (degranulating and/or secreting multiple cytokines; 2, 3, 4, or 5 functions) were identified using the Boolean gating strategy available at FlowJo v10 software. (B) For the phenotype panel, the initial gating strategy was identical to the polyfunctionality panel up-to-the point of CD8 vs. CD4 plot. There, CD8+ events were gated to define bulk CD8+ T-cells and a CD8 vs. FITC plot was derived to identify HIV-specific CD8+ T-cells (defined as the ones degranulating and/or expressing cytokines, all stained in FITC). Subsequent analyses were performed on both populations as shown by overlaid dot-plots and overlaid histograms. To analyze the distribution of the different phenotype subsets, CD45RO vs. CCR7 density plots were constructed to identify central memory T-cells (TCM, CCR7+/CD45RO+), effector memory T-cells (TEM, CCR7−/CD45RO+) and terminal effector T-cells (TTE, CCR7−/CD45RO−). CD95 expression was analyzed within the CD45RO−CCR7+ cells thus defining naïve T-cells (TN, CCR7+/CD45RO−/CD95−) and stem-cell memory T-cells (TSCM, CCR7+/CD45RO−/CD95+). Additionally, PD-1 e [file Image_1.JPEG]

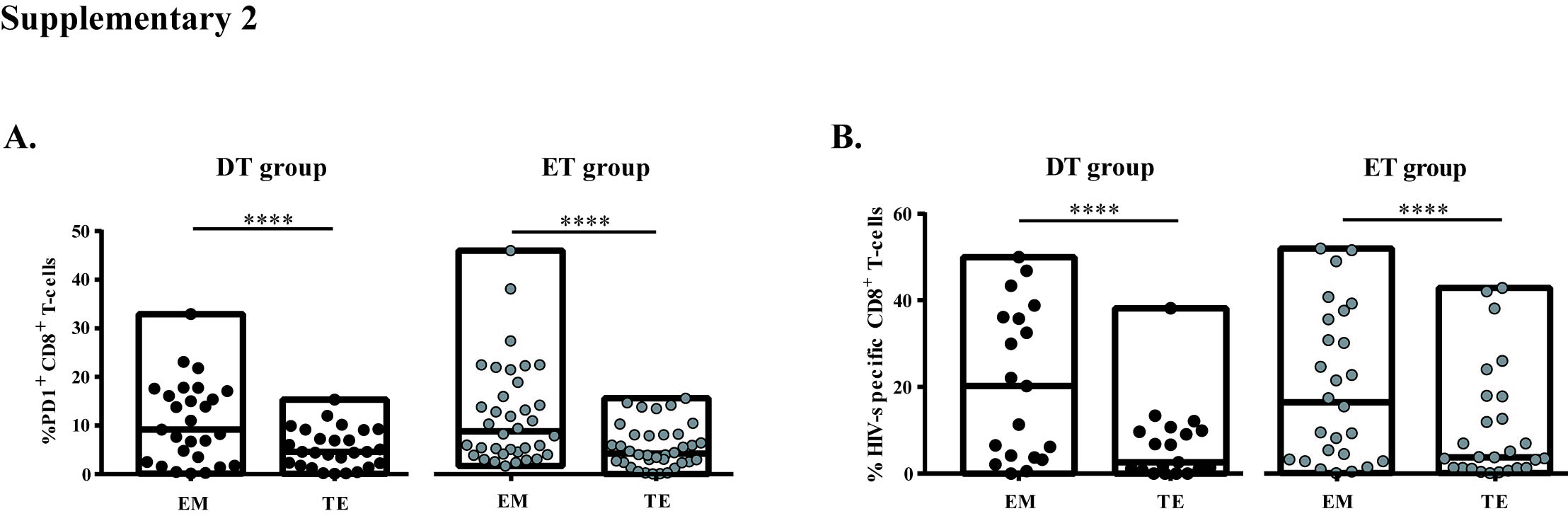

Supplement: Figure S2 — (A) Proportion of PD-1+ cells observed post-expansion on bulk CD8+ TEM and TTE cells from DT and ET individuals. (B) Proportion of HIV-specific cells (either Nef-specific or p24-specific) cells, identified on the bases of cytokine production and/or degranulation capacity, observed post-expansion on CD8+ TEM and TTE cells from DT and ET individuals. In (A,B), boxes extend from min to max. Horizontal bar within boxes represent the median. ****p ≤ 0.0001 according to Wilcoxon's test. [file Image_2.JPEG]
